# Clinical and ultrasound characteristics of virilizing ovarian tumors in pre- and postmenopausal patients: a single tertiary center experience

**DOI:** 10.1186/s13023-021-02057-z

**Published:** 2021-10-12

**Authors:** Mi Zou, Rong Chen, Yahong Wang, Yonglan He, Ying Wang, Yifan Dong, Jianchu Li

**Affiliations:** 1 Department of Ultrasound, State Key Laboratory of Complex Severe and Rare Diseases, Peking Union Medical College Hospital, Chinese Academy of Medical Science and Peking Union Medical College, 1 Shuaifuyuan, Dongcheng District, Beijing, 100730 China; 2 Department of Obstetrics and Gynecology, Peking Union Medical College Hospital, Beijing, China; 3 Department of Radiology, Peking Union Medical College Hospital, Beijing, China

**Keywords:** Virilizing ovarian tumor, Hyperandrogenism, Ultrasound

## Abstract

**Background:**

A virilizing ovarian tumor (VOT) is a rare cause of hyperandrogenism in pre- and postmenopausal women. Although transvaginal ultrasound is considered as the first-line imaging method for ovarian tumors, it is examiner-dependent. We aimed to summarize the clinical and ultrasound manifestations of VOTs to help establish the diagnosis with emphasis on those causing diagnostic difficulty.

**Method:**

We retrospectively identified 31 patients with VOTs who underwent surgery at Peking Union Medical College Hospital.

**Results:**

Patients with VOTs were predominantly premenopausal. All patients showed androgenic manifestations with serum testosterone levels elevated to varying degrees. The tumor size of VOTs was significantly correlated with age (*P* < 0.001). The VOTs in the postmenopausal group were significantly smaller than those in the premenopausal group (median 1.8 cm [range, 1.3–4.8 cm] vs 4.5 cm [range, 0.7–11.9 cm]; *P* = 0.018). Twenty-seven out of 31 VOTs were successfully identified by ultrasound. On ultrasound, VOTs are mostly solid and hypoechoic masses with enhanced vascularity. Four VOTs (0.7–1.5 cm) were radiologically negative, and they were the smallest among all patients.

**Conclusion:**

Patients with VOTs showed androgenic manifestations with varying degrees of hyperandrogenemia. Older patients tend to have smaller VOTs. Ultrasound is an effective method for the detection of VOTs. Some VOTs can be very small and difficult to visualize radiologically, especially in postmenopausal patients. Examiners must remain vigilant about very small VOTs on the basis of endocrine symptoms.

## Introduction

Virilizing ovarian tumors (VOTs) are uncommon, accounting for less than 1% of all ovarian tumors and less than 0.2% of all cases of hyperandrogenism in women [1, 2]. VOTs should be considered in the context of the rapid pace of development of hirsutism or signs of virilization (i.e., alopecia, a deepened voice, clitoromegaly, increased muscle mass) [3]. Laboratory tests are required to confirm androgen excess, and radiological studies are needed to identify the source of the secretion. Pelvic ultrasound is the first-line imaging method for identifying VOTs, and second-line radiological assessments, including contrast-enhanced MRI and PET-CT, can be helpful when ultrasound is not sufficiently revealing. Sometimes even with multiple radiological methods, identifying the source of the excess androgen and establishing the diagnosis of VOTs can be difficult [4]. A precise preoperative location of VOTs can lead to minimally invasive treatment, including ovarian tumor removal or unilateral salpingo-oophorectomy instead of bilateral salpingo-oophorectomy in some radiologically negative cases, which is important for preservation of ovarian function and fertility. The existence of differences in clinicopathological and ultrasound features of VOTs in pre- and postmenopausal patients has been speculated by physicians based on individual cases but seldom compared in published studies due to its rarity. Thus, a greater understanding of the clinical and imaging characteristics of VOTs is needed. The purpose of the study was to analyze the clinical and imaging manifestations of VOTs to help establish the diagnosis with emphasis on those causing diagnostic difficulty.

## Materials and methods

Following approval by the Internal Review Board of Peking Union Medical College Hospital, we performed a retrospective study of hospitalized patients with VOTs confirmed by surgery and pathological examination from 2012 to 2019. Thirty-one patients with VOTs were enrolled in the study. The levels of serum testosterone were measured using a chemiluminescence assay on the Beckman DXI800 platform (reference range for adult women, 0.10–0.75 ng/ml). Patient medical records were reviewed, and the following relevant clinical information was recorded: age, clinical symptoms (signs of virilization, menstrual abnormalities), hormonal levels, radiological features, pathological results and follow-up data. The patients were divided into premenopausal and postmenopausal groups. Menopausal status (defined as the absence of menses for greater than 1 year) was confirmed by a medical record review.

### Statistical analysis

Categorical variables are stated in actual numbers and percentages. Continuous variables were described as the median (range, minimum–maximum). Categorical variables between two groups were compared by using Fisher’s exact test or the χ^2^ test. Continuous variables were compared by using the independent t-test, Mann–Whitney U test or Kruskal–Wallis test. Correlations between two variables were calculated using Pearson’s correlation coefficient (r). A *P* value < 0.05 was considered statistically significant. Statistical analysis was performed with SPSS software version 20.0 (IBM SPSS, Chicago, Illinois, USA).

## Results

### Clinicopathologic features

A total of 31 patients with VOTs were included in the study, among whom 23 (74.2%) were premenopausal and eight (25.8%) were postmenopausal. The comparison of clinicopathologic features between these two groups is presented in Table 1.

**Table 1:** Clinicopathological features of 31 patients with VOTs

Characteristics	All (n = 31)	Premenopausal (n = 23)	Postmenopausal (n = 8)	*P* value
Age	34 (14–66)	30 (14–52)	60 (56–66)	
Presenting symptom				
Hirsutism	28 (90.3)	20 (87)	8 (100)	0.550
Alopecia	6 (19.4)	3 (13)	3 (37.5)	0.161
Acne	6 (19.4)	5 (21.7)	1 (12.5)	1.000
Deepened voice	11 (35.5)	7 (30.4)	4 (50)	0.405
Clitoromegaly	15 (48.4)	12 (52.2)	3 (37.5)	0.685
Symptom duration (months) (range)	36 (6–168)	36 (12–168)	27 (6–132)	0.317
Testosterone level (ng/ml) (range)	3.6 (1.2–16.2)	3.6 (1.2–14.4)	3.7 (1.9–16.4)	0.74
Tumor diameter (cm) (range)	3.1 (0.7–11.9)	4.5 (0.7–11.9)	1.8 (1.3–4.8)	0.018*
Histotype				
Sertoli-Leydig cell tumors	9 (29.0)	9 (39.1)	–	0.041*
Leydig cell tumors	9 (29.0)	3 (13)	6 (75)	0.010*
Steroid cell tumors	7 (22.6)	6 (26.1)	1 (12.5)	0.015*
Granulosa cell tumors	4 (12.9)	3 (13)	1 (12.5)	1.000
Sclerosing stromal tumor	1 (3.2)	1 (4.3)	–	
Sex cord tumor with annular tubules	1 (3.2)	1 (4.3)	–	

Data are given as n or n (%)

Asterisks (*) indicate statistically significant differences between the two groups (*P < 0.05 and **P < 0.01)

All patients presented with androgenic manifestations, of which hirsutism was the most common presenting symptom. All patients of reproductive age had oligomenorrhea or amenorrhea. All postmenopausal patients presented with clinical signs of hyperestrogenism, including postmenopausal hemorrhage and thickened endometrial lining. There was no case with associated ascites. The median interval between the onset of symptoms as perceived by patients and the surgical removal of the VOT was 36 months (range, 6–168 months). The median serum testosterone level was 3.6 ng/ml (range, 1.2–16.2 ng/ml). Comparative analysis showed no significant differences in presenting symptoms, symptom duration or testosterone levels between the two groups.

**Fig. 1:**
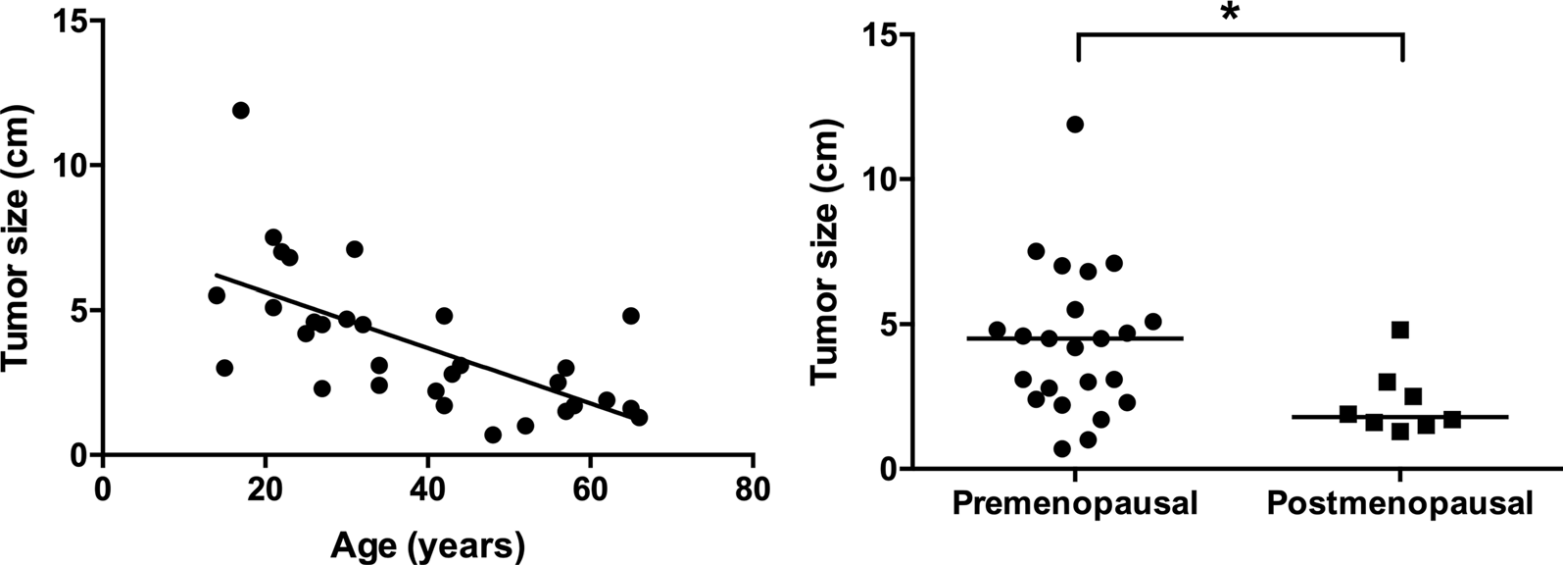
**a** Correlation between age and tumor size. Tumor size was correlated with age (correlation coefficient, r = 0.42, tumor size = 0.096 × age + 7.540, *P* < 0.001). **b** Individual values of the sizes of the VOTs in the premenopausal and postmenopausal groups. A significant difference between the two groups was observed (*P* = 0.018)

All the tumors were unilateral. The median tumor size was 3.1 cm (range, 0.7–11.9 cm). A significant correlation between tumor size and age was observed (*P* < 0.001) (Fig. 1a). The tumor size was significantly smaller in the postmenopausal group than in the premenopausal group (median 1.8 cm [range, 1.3–4.8 cm] vs 4.5 cm [range, 0.7–11.9 cm]; *P* = 0.018) (Fig. 1b).

Histological examination showed nine (29.0%) Sertoli-Leydig cell tumors, nine (29.0%) Leydig cell tumors, seven (22.6%) steroid cell tumors, four (12.9%) granulosa cell tumors, one (3.2%) sclerosing stromal tumor and one (3.2%) sex-cord tumor with annular tubules. One (11.1%) of the Sertoli-Leydig cell tumors was well differentiated, five (55.6%) were intermediately differentiated, and three (33.3%) were poorly differentiated. In the premenopausal group, the most common VOTs were Sertoli-Leydig cell tumors (39.1%). The most frequent VOTs in the postmenopausal group were Leydig cell tumors (75.0%). Sertoli-Leydig cell tumors were significantly more common in premenopausal patients than in postmenopausal patients (39.1% vs 0%, *P* = 0.041). Leydig cell tumors were significantly more common in postmenopausal patients than in premenopausal patients (75.0% vs 13.0%, *P* = 0.010).

**Fig. 2:**
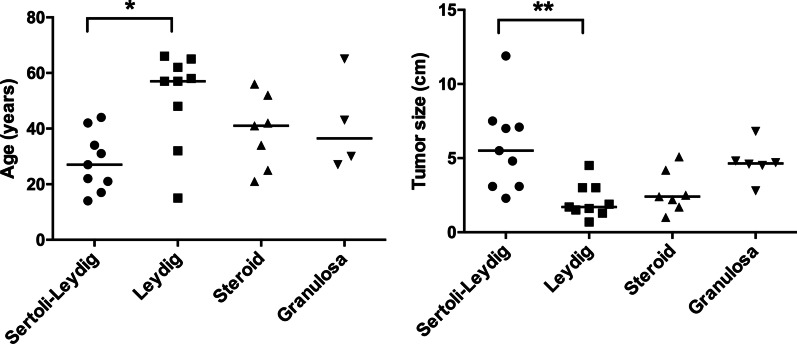
Individual values of age (**a**) and size (**b**) for the cases with Sertoli-Leydig cell tumors, Leydig cell tumors, steroid cell tumors and granulosa cell tumors. Significant differences in age and size between Sertoli-Leydig cell tumors and Leydig cell tumors were observed (adjusted *P* = 0.021, 0.003, respectively)

Age and tumor size were significantly different between the four most frequent histotypes (*P* = 0.034 and 0.003, respectively) (Fig. 2). The patients with Leydig cell tumors were significantly older than those with Sertoli-Leydig cell tumors (median 57 years [range, 15–66 years] vs 27 years [range, 14–44 years]; *P* = 0.021). The sizes of Leydig cell tumors were significantly smaller than the sizes of Sertoli-Leydig cell tumors (median 1.7 cm [range, 0.7–4.5 cm] vs 5.5 cm [range, 2.3–11.9 cm], *P* = 0.003).

### Imaging evaluation

In this cohort, all patients underwent ultrasound examinations before other pelvic radiological examinations. Because transvaginal ultrasound (TVUS) allows for optimal visualization of the ovary by providing higher-quality images than transabdominal ultrasound (TAUS), patients suspected of having VOTs underwent TVUS unless they had contraindications, including virgins and vaginal obstruction. TVUS revealed 22 ovarian tumors (median, 3.1 cm; range, 1.6–7.1 cm) in 26 patients. TAUS detected all ovarian tumors (median, 5.5 cm; range, 3.0–11.9 cm) in five patients. In total, specific ovarian lesions were revealed in 27 out of 31 patients by ultrasound examinations and reconfirmed by other radiological examinations in some cases. While the other four ovarian lesions were radiologically negative, multiple radiological examinations, including repeated TVUS and one or two other radiological examinations, including contrast-enhanced computed tomography (CT), contrast-enhanced MRI or FDG PET-CT scans, failed to reveal the ovarian tumors.

### Ultrasound manifestations

**Fig. 3:**
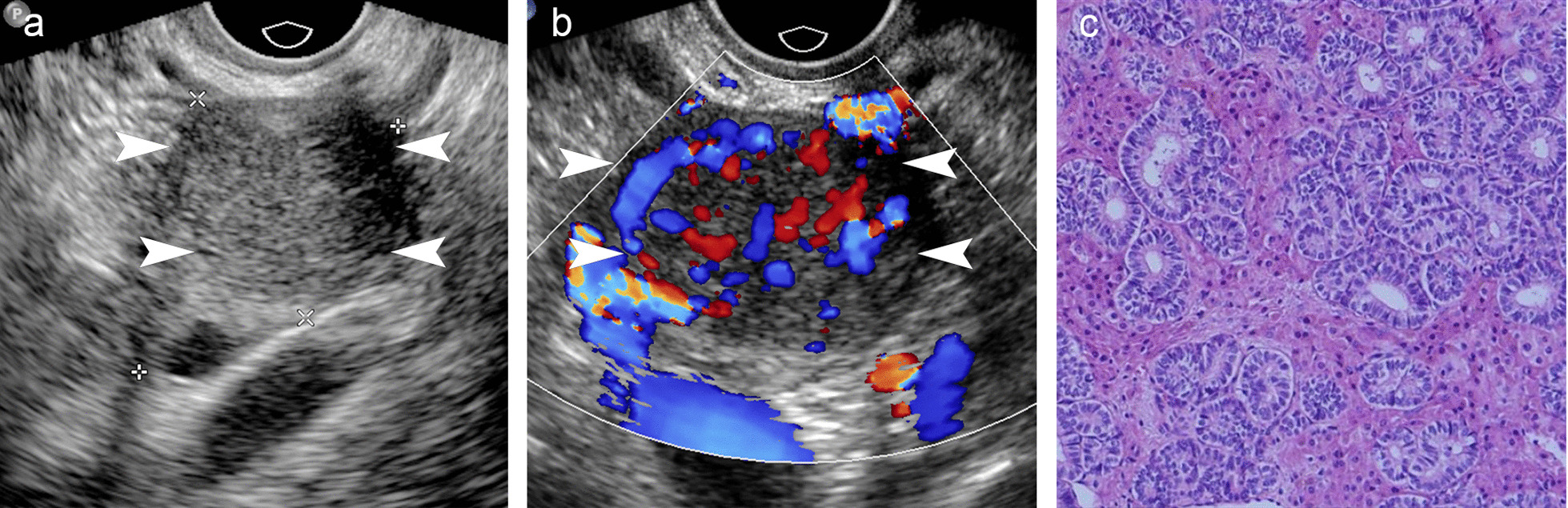
Grayscale, color Doppler ultrasound and microscopic images of a small solid Sertoli-Leydig tumor. **a** Left ovary with a small isoechogenic solid tumor (arrowhead). **b** Ultrasound with color Doppler in the transverse plane reveals abundant blood flow signals (arrowhead). **c** Histopathology showing tubules composed of Sertoli cells with interspersed small clusters of Leydig cells

**Fig. 4:**
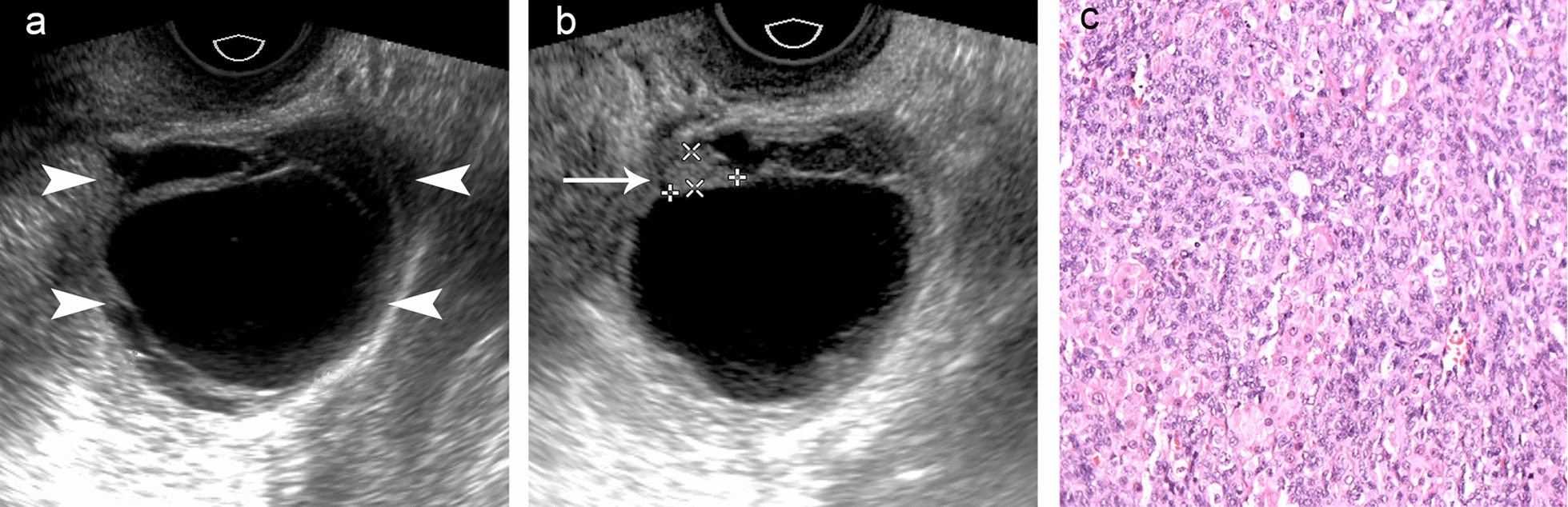
Grayscale ultrasound (**a**, **b**) and microscopic images (**c**) of a cystic granulosa cell tumor with septa (arrowhead) and a papillary projection (arrow)

In the ultrasound study, tumor sizes ranged from 1.6 to 11.9 cm (median, 4.2 cm). Twenty-one (77.8%) tumors were solid (Fig. 3), and six (22.2%) tumors had both solid and cystic components (Fig. 4). The solid component of 18 tumors was hypoechoic, seven were isoechoic, one was hyperechoic, and one had calcification. Color Doppler flow imaging showed vascularity in all 27 masses and relatively abundant vascularity in 17 masses.

### MRI and PET-CT manifestations

**Fig. 5:**
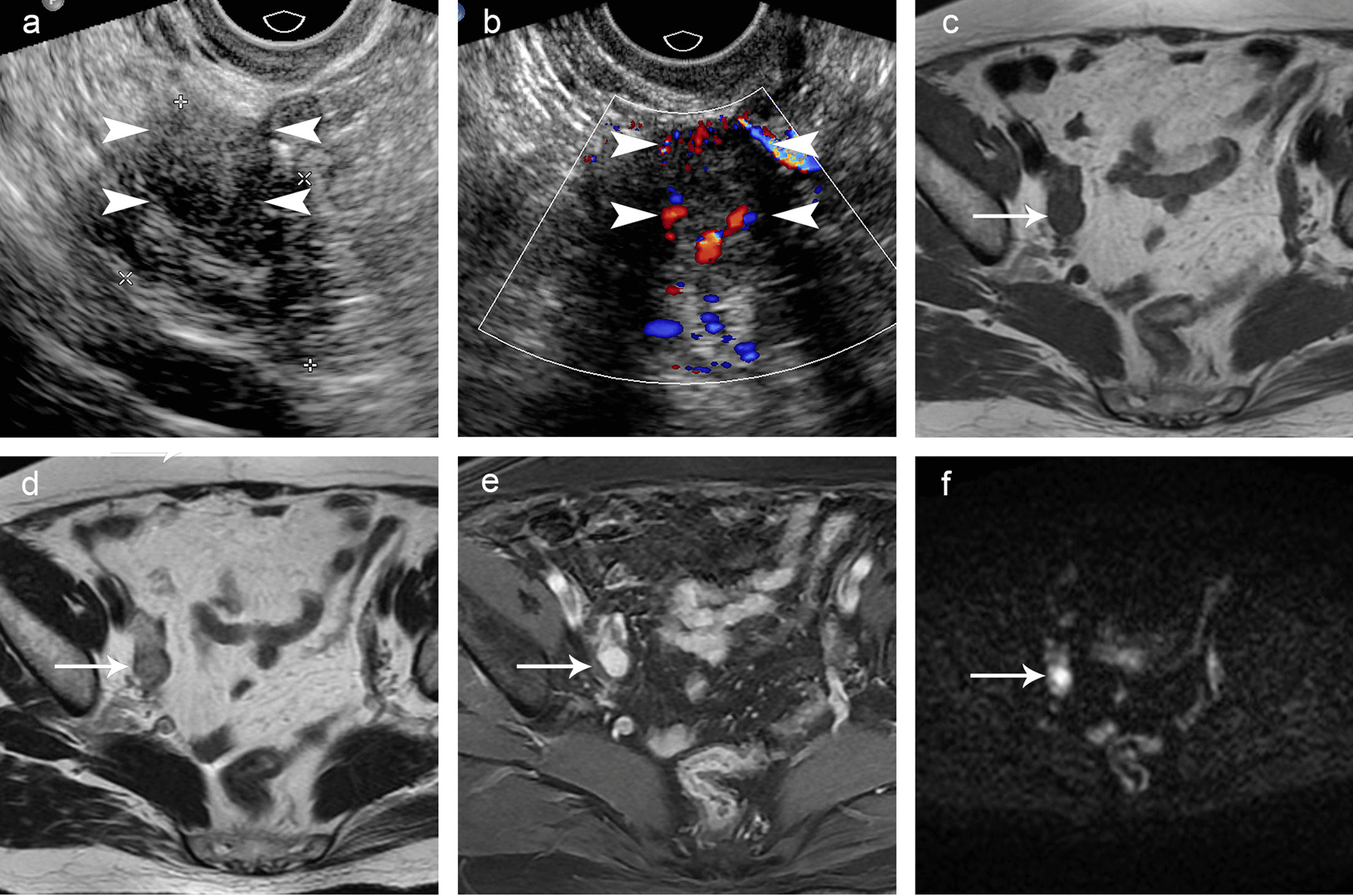
Ultrasound, MRI and microscopic images of a 58-year-old patient with a small Leydig cell tumor. The right ovarian volume was more than twice that of the opposite side, with the right measuring 3.4 × 1.8 × 2.3 cm (volume 7.0 cm^3^) and the left measuring 2.3 × 1.1 × 1.0 cm (volume 1.3 cm^3^). The right ovary had a slightly hypoechoic area (arrowhead) on grayscale ultrasound (**a**), which was delineated with a relatively rich blood flow signal on Doppler ultrasound (arrowhead, **b**). Contrast-enhanced MRI in axial view showing a small lesion, which is a slightly hypointense signal in the T1-weighted images (arrow, **c**), a heterogeneous hyperintense signal in the T2-weighted images (arrow, **d**), a heterogeneous hyperenhanced signal in the gadolinium-enhanced T1-weighted images (arrow, **e**), and a partially hyperintense signal in the diffusion weighted images (DWI) (b = 800) (arrow, **f**)

Ten patients underwent contrast-enhanced MRI, and the results revealed six specific lesions, which were consistent with the ultrasound findings (Fig. 5). The MRI appearance of these tumors varies with their morphologies and components but shares the commonality that the VOTs had intense enhancement, reflecting the rich vascularity of the tumor. Two TVUS-positive VOTs eluded contrast-enhanced MRI. Four patients had PET-CT scans with one ovarian tumor identified by increased uptake. Two TVUS positive VOTs eluded PET-CT scans.

### Radiologically negative patients

**Fig. 6:**
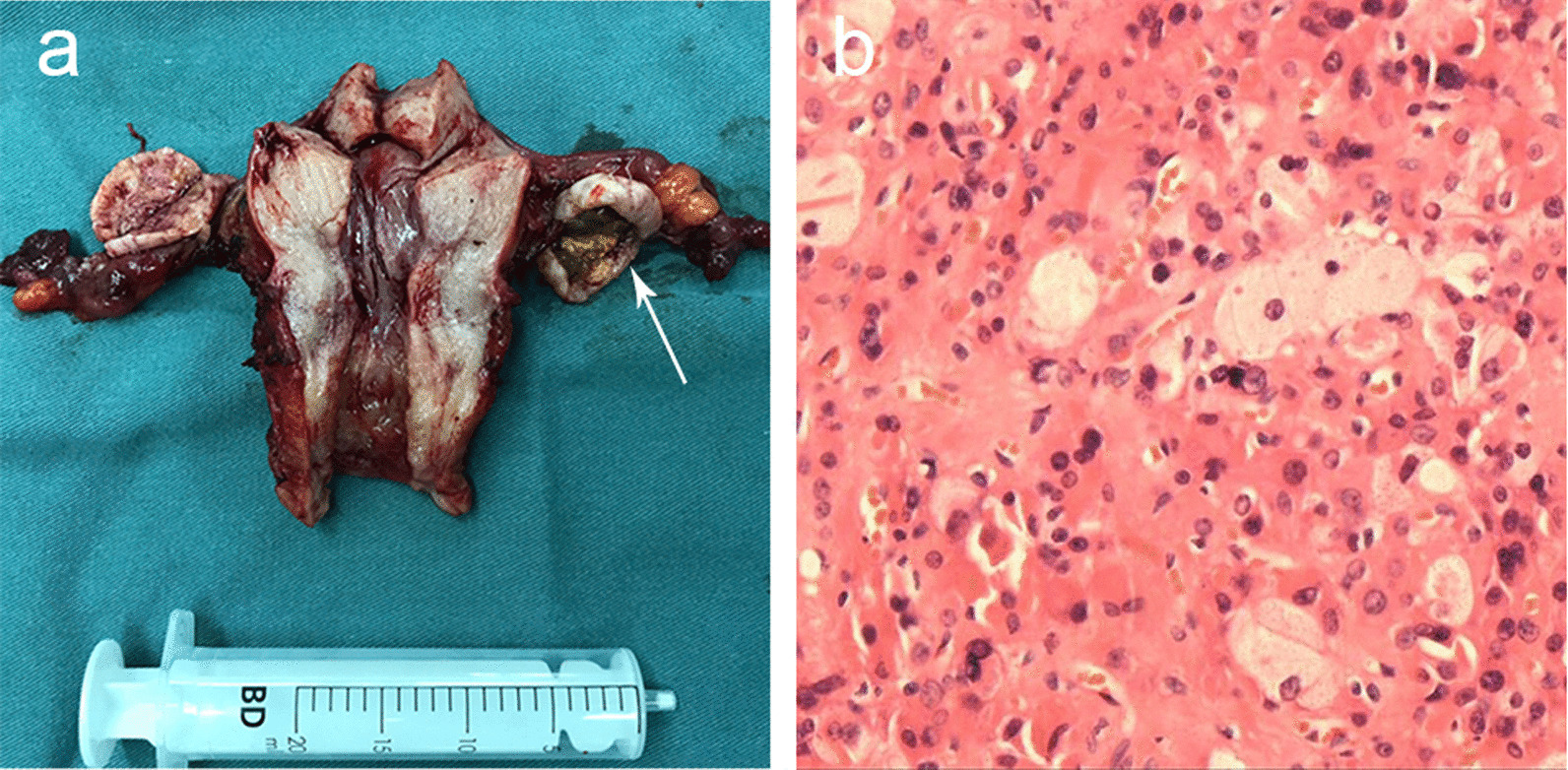
**a** Gross inspection showing that the cut surface of an ultrasound-negative left ovary in a 66-year-old patient manifested a central yellow solid part with a peripheral grayish green solid part (arrow). **b** At histological examination (400X), the lesion was found to be a Leydig cell tumor

Two of the four radiologically negative patients were postmenopausal, and the other two were premenopausal, aged 48 and 52 years. The median testosterone level was 3.8 ng/ml (range, 3.1–14.4 ng/ml). The dehydroepiandrosterone sulfate (DHEA-S) and 17-hydroxyprogesterone (17-OHP) levels were within the normal range. Dexamethasone suppression tests (0.75 mg, 4 times a day for 5 consecutive days) were performed in these four patients and revealed unsuppressed testosterone levels. Laboratory results were not indicative of an adrenal source of androgens. In two patients, the ovaries with VOTs could not be visualized by TVUS. However, in the other two patients, the ovaries appeared to be normal, and VOTs were not identified by TVUS. The following second-line radiological examinations did not achieve proper radiological identification of these four ovarian tumors either, and preoperative localization of the origin of the excessive androgen was mainly based on laboratory results without the support from conclusive radiological findings. Because of the strong suspicion of VOTs and the lack of a need for fertility preservation in these four patients, diagnostic and surgical laparoscopy was performed on each patient without selective ovarian vein sampling. During laparoscopy, the disclosed ovaries were seemingly of regular shape and normal size. Hysterectomy and bilateral salpingo-oophorectomy were performed. Gross inspection showed lesions on the ovaries of the four patients (Fig. 6), with the size for each patient being 0.7 cm, 1 cm, 1.3 cm and 1.5 cm, and these tumors were the smallest four tumors among all patients in the cohort. Histological examination revealed three Leydig cell tumors and one steroid cell tumor.

### Treatment and follow-up

All patients underwent surgical treatment, and three had adjuvant chemotherapy for poorly differentiated tumors. In the premenopausal group, 18 patients underwent unilateral salpingo-oophorectomy, three underwent ovarian tumor removal, and two underwent hysterectomy and bilateral salpingo-oophorectomy. In the postmenopausal group, all patients underwent hysterectomy and bilateral salpingo-oophorectomy. Serum testosterone levels were retested in these patients within 1 month of surgical treatment, and all were within the normal range. Follow-up information was available for 26 patients, with a median follow-up duration of 52.3 months (range, 26.4–103.2 months). These patients were all alive without recurrent disease at the end of the follow-up period.

## Discussion

VOTs are uncommon neoplasms that affect patients within a wide range of ages and are mainly present in premenopausal patients. Our study showed that patients with different menopausal statuses had similar presentations of virilizing symptoms and equally elevated levels of testosterone. Ultrasound showed high sensitivity in detecting VOTs. However, postmenopausal patients tend to have smaller tumors than premenopausal patients, and diagnosing very small VOTs can be challenging even when multiple radiological imaging methods are used. The primary treatment for VOTs was surgery, and the prognosis was often very favorable.

The testosterone levels associated with VOTs that we typically see in our clinic are > 1.2 ng/mL, which is in agreement with the previous literature that a circulating testosterone value of 1.50 ng/mL has been considered a reasonable criterion to discriminate women with benign forms of hyperandrogenism from those with suspected ovarian malignant disease [5].

Our study is the first to demonstrate that VOTs are significantly smaller in postmenopausal women than in premenopausal women. In accordance with a previous report [6], the most common VOT was Sertoli-Leydig cell tumors. The present study revealed that the most common histotypes differed between premenopausal and postmenopausal patients, with Sertoli-Leydig cell tumors in premenopausal patients and Leydig cell tumors in postmenopausal patients. The sizes of Leydig cell tumors were significantly smaller than the sizes of Sertoli-Leydig cell tumors. The distinct size and peak incidence of Sertoli-Leydig cell tumors and Leydig cell tumors may be the reason for the difference in the size of VOTs between pre- and postmenopausal patients. This feature makes the topological diagnosis of very small VOTs challenging, which often occurs in postmenopausal patients.

In general, ultrasound allowed adequate tumor detection in our study. In this study, the smallest VOT detected by TVUS was 1.6 cm, and the four VOTs that were smaller than 1.6 cm were overlooked by TVUS. A VOT could elude TVUS due to small tumor size, isoechogenic appearance to the surrounding ovarian tissue, failure of visualization of the ovarian and misinterpreted ultrasound findings. Two VOTs measured slightly smaller than 1.5 cm have been reported to be successfully identified using TVUS, and they were either hypoechoic or hyperechoic on TVUS [7, 8]. The level of expertise of the ultrasound examiners varied. A comprehensive understanding and well-established ultrasound skills allow for better detection of VOTs. VOTs are mostly unilateral, solid and hypoechoic masses with enhanced vascularity. Ultrasound examiners must remain cautious about very small VOTs on the basis of endocrine symptoms, especially in postmenopausal patients, because they tend to have smaller VOTs. Regarding VOTs in postmenopausal women, an elevated testosterone level is often found and confirmed by clinical history; sonographers must attempt to identify a hypothetical small tumor in atrophied ovaries, which are not easy to visualize. After the onset of menopause, the ovary atrophies rapidly over subsequent years, with the disappearance of follicles and a decrease in ovarian volume; thus, the ovary may be difficult to visualize by TVUS or MRI. The normal morphology of ovaries cannot be used to exclude VOTs because these tumors can be so small that they are well contained even within postmenopausal ovaries. Ultrasound measurement of ovarian volume is reported to assist physicians in the early diagnosis of ovarian neoplasia [9]. Normal ovarian volumes fell within a predictable range (3.4 ± 1.7 cm^3^), with an upper limit of normal at 8.0 cm^3^ for postmenopausal women [10]. When asymmetrical ovaries are found and the grayscale ultrasound shows an ambiguous lesion, color Doppler imaging is crucial for the detection of abnormal vascularity, indicating insidious tumors.

In our study, second-line radiological modalities, including contrast-enhanced MRI and PET-CT, did not confirm the presence of VOTs in the four ultrasound-negative cases. Sarfati et al. [11] reported a higher positive predictive value and negative predictive value for MRI than for ultrasound VOTs in postmenopausal patients, but the authors did not provide any imaging details, particularly whether it was TAUS or TVUS that was used in the study and the sizes of their three MRI-positive tumors. Because of the effects of intestinal gas and the abdominal wall, it is possible that small VOTs elude TAUS, and as Fanta et al. [12] demonstrated in three cases, it is very easy for a small tumor to be overlooked or interpreted as a different gynecological pathology if TVUS is not performed by an experienced examiner. 18F-FDG-PET imaging revealed only one out of four VOTs in our study. There are some cases successfully using 18F-FDG-PET imaging to identify androgen-secreting tumors [13–17], but such cases are limited. There is another case of VOT that was not identified on 18F-FDG-PET imaging but was identified on ^11^C-acetate-PET [18]. Therefore, the utility of 18F-PET-CT for the identification of VOTs is mild. When conventional imaging techniques such as TVUS and MRI are inconclusive, PET-CT may be useful as an adjunctive tool. For patients suspected of having VOTs clinically and biochemically, when imaging studies are not revealing, clinical decision making requires careful consideration of menopausal status. Invasive methods, including selective ovarian vein sampling and exploratory laparoscopy, could possibly enable the identification of VOTs and lead to minimally invasive treatment to serve the need for fertility preservation. For postmenopausal patients, exploratory laparoscopy and bilateral salpingo-oophorectomy could meet the diagnostic and treatment needs.

This study is mainly limited by its retrospective nature and single-center design. Therefore, these findings should be interpreted with caution. Another limitation, because the study population was restricted to hospitalized patients who underwent ovarian surgery in a tertiary hospital, is the findings of this study may not be extrapolated to general clinical settings.

## Conclusion

Patients with VOTs showed androgenic manifestations with varying degrees of hyperandrogenemia. Older patients tend to have smaller VOTs. Ultrasound is an effective method for the detection of VOTs. Some VOTs can be very small and difficult to visualize radiologically, especially in postmenopausal patients. Examiners must remain vigilant about very small VOTs on the basis of endocrine symptoms.

## Data Availability

The datasets generated and/or analyzed during the current study are available from the corresponding author on reasonable request.
